# [Corrigendum] FPR2 serves a role in recurrent spontaneous abortion by regulating trophoblast function via the PI3K/AKT signaling pathway

**DOI:** 10.3892/mmr.2023.12998

**Published:** 2023-04-18

**Authors:** Anna Li, Shuxian Li, Chongyu Zhang, Zhenya Fang, Yaqiong Sun, Yanjie Peng, Xietong Wang, Meihua Zhang

Mol Med Rep 24: 838, 2021; DOI: 10.3892/mmr.2021.12478

Subsequently to the publication of the above paper, an interested reader drew to the authors’ attention that the ‘0 h/si-NC + Solvent’ and ‘0 h/si-FPR2 + Solvent’ data panels shown in Fig. 4B on p. 7 appeared to contain overlapping sections of data, such that they were potentially derived from the same original source where these panels was intended to show the results from differently performed experiments. After having conducted an independent investigation of the figures in the Editorial Office, it was identified that sections of the ‘si-NC + Solvent’ and ‘si-FPR2 + LY294002’ data panels in Fig. 4C also contained overlapping data.

After having asked the authors to provide an explanation of these data, they realized that this figure was inadvertently assembled incorrectly. They were, however, able to consult their original data, and the revised version of Fig. 4, containing the correct data panel for the ‘si-FPR2 + LY294002’ experiment in Fig. 4C and complete data from one of the alternatively performed experiments in Fig. 4B, is shown on the next page. Note that these errors did not adversely affect either the results or the overall conclusions reported in this study. All the authors agree with the publication of this corrigendum, and are grateful to the Editor of *Molecular Medicine Reports* for allowing them the opportunity to publish this. They also wish to apologize to the readership of the Journal for any inconvenience caused.

## Figures and Tables

**Figure 4.**
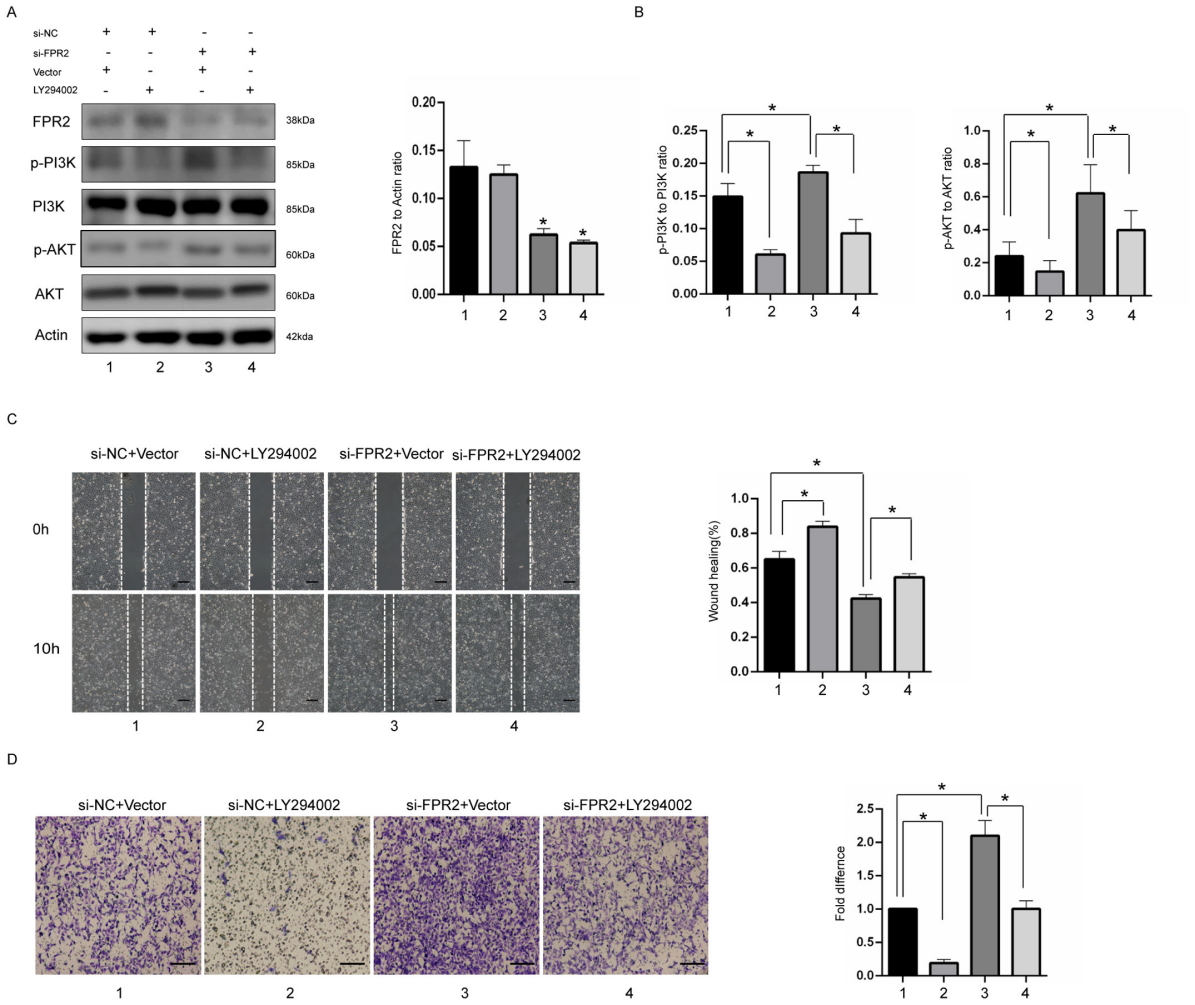
Knockdown of FPR2 promotes cell migration and invasion through the PI3K-AKT pathway. (A) Western blotting of FPR2, p-PI3K, PI3K, p-AKT and AKT in HTR8 cells transfected with si-FPR2 or si-NC in the presence or absence of LY294002 and densitometry quantification of western blots. (B) Gap closure and (C) Transwell assays of FPR2 silencing cells in the presence of LY294002. Scale bars=200 µm. Data are presented as mean ± SD obtained from at least three independent experiments. *P<0.05, **P<0.01. FPR2, formyl peptide receptor 2; p-, phosphorylated; si, small interfering; NC, negative control..

